# Identification of Genetic Markers Linked to The Activity of Indoleamine 2,3-Dioxygenase and Kidney Function

**DOI:** 10.3390/metabo13040541

**Published:** 2023-04-10

**Authors:** Hye-Rim Kim, Hyun-Seok Jin, Yong-Bin Eom

**Affiliations:** 1 Department of Medical Sciences, Graduate School, Soonchunhyang University, Asan 31538, Chungnam, Republic of Korea; goa6471@naver.com; 2 Department of Biomedical Laboratory Science, College of Life and Health Sciences, Hoseo University, Asan 31499, Chungnam, Republic of Korea; jinhs@hoseo.edu; 3 Department of Biomedical Laboratory Science, College of Medical Sciences, Soonchunhyang University, Asan 31538, Chungnam, Republic of Korea

**Keywords:** indoleamine 2,3-dioxygenase (IDO), chronic kidney disease (CKD), estimated glomerular filtration rate (eGFR), single nucleotide polymorphism (SNP), genome-wide association study (GWAS)

## Abstract

Indoleamine 2,3-dioxygenase (IDO) is a tryptophan-degrading enzyme belonging to the kynurenine pathway. IDO activity has been suggested as a potential biomarker for early diagnosis of chronic kidney disease (CKD). The aim of this study was to perform coincident association analysis to gain genetic insights into the correlation between IDO activity and CKD. This study evaluated the association between IDO activity and CKD using the Korea Association REsource (KARE) cohort. Logistic and linear regression were used to analyze CKD and quantitative phenotypes such as IDO and estimated glomerular filtration rate (eGFR). Our results identified 10 single nucleotide polymorphisms (SNPs) that were coincidently associated with both IDO and CKD (*p* < 0.001). Among them, rs6550842, rs77624055, and rs35651150 were selected as potential candidates after excluding SNPs with insufficient evidence for having an association with IDO or CKD. Further expression quantitative trait loci (eQTL) analysis for variants at selected loci showed that rs6550842 and rs35651150 significantly affected the expression of *NKIRAS1* and *SH2D4A* genes in human tissues, respectively. Additionally, we highlighted that the *NKIRAS1* and *BMP6* genes were correlated with IDO activity and CKD through signaling pathways associated with inflammation. Our data suggest that *NKIRAS1*, *SH2D4A*, and *BMP6* were potential causative genes affecting IDO activity and CKD through integrated analysis. Identifying these genes could aid in early detection and treatment by predicting the risk of CKD associated with IDO activity.

## 1. Introduction

Chronic kidney disease (CKD) is a worldwide public health problem, which can lead to a gradual decline in kidney function [1]. Diabetes and high blood pressure are among the primary factors responsible for its roughly [2]. According to a recent Center for Disease Control and Prevention (CDC) report, about 15% of American adults suffer from CKD, with many of them being oblivious to their condition [3]. The Korea Disease Control and Prevention Agency (KDCA) also reported that the prevalence of CKD among Korean adults in 2019 was 9.3% (https://health.kdca.go.kr/ (accessed on 10 March 2023)). Continued kidney damage can lead to end-stage renal disease (ESRD) requiring dialysis or kidney transplantation or increase the risk of cardiovascular complications [2]. Therefore, early identification and effective control of CKD are important to prevent these diseases [2,4]. Creatinine and estimated glomerular filtration rate (eGFR) are mainly used as indicators of kidney damage [5]. However, they are insufficiently sensitive to predict CKD at an early stage [6]. Thus, further studies are needed to identify novel biomarkers.

Indoleamine 2,3-dioxygenase (IDO) is a rate-limiting enzyme of tryptophan metabolism that catalyzes the degradation of tryptophan to kynurenine [7]. Its activity is induced by pro-inflammatory cytokines such as interferon-γ (IFN-γ) and tumor necrosis factor-α (TNF-α) [8]. Previously published reports have demonstrated a correlation between some types of renal disease and IDO activity [9,10,11,12]. In detail, IDO expression was found in the glomerular and tubular cells of the nephrotoxic serum nephritis model [9] and increased apoptosis in tubular cells of the renal ischemia-reperfusion injury model [10], respectively. IDO was also associated with diabetic nephropathy [12] and renal fibrosis [11]. Furthermore, several studies have demonstrated the association between CKD and IDO activity [7,13,14,15].

Renal inflammation is an initial response to kidney damage, but if inflammation persists, it promotes the fibrotic process, leading to CKD [16]. Interestingly, Matheus et al. showed that IDO accompanies renal fibrosis, and its expression is induced by transforming growth factor-β (TGF-β) 1, a potent fibrotic molecule [11]. Another study demonstrated that IDO activity is associated with CKD and key inflammatory markers (hs-CRP, high-sensitivity C-reactive protein, and sTNFR-I, soluble TNF-receptor-1) [13]. The precise mechanism of IDO in CKD remains uncertain. Nevertheless, the above findings suggest that the correlation between CKD and IDO may be mainly induced by inflammatory mechanisms.

To better understand these complex diseases, recent studies have used an integrated analysis of omics, including genomics, transcriptomics, proteomics, metabolomics, and epigenomics [17,18]. CKD has been proven to be a complex disease with high heritability [2,19]. Hence, we hypothesized that genetic factors might play a role in altering IDO activity and through a combining genomics and metabolomics data analysis of a Korean cohort, we have recently identified multiple loci linked to IDO activity associated with CKD [15]. However, since our previous study had a narrow focus on IDO activity, the statistical significance of CKD and eGFR was paid less attention. To compensate for this and to find genes coincidentally associated with CKD and IDO activity, we broadened the statistical significance to 0.01. Additionally, loci affecting mRNA expression were then identified through expression quantitative trait loci (eQTL) analysis among variants related to CKD and IDO activity.

## 2. Materials and Methods

### 2.1. Study Population

The Korea Association REsource (KARE) cohort used in this study has been described in detail elsewhere [20]. Briefly, the KARE cohort as part of the Korean Genome and Epidemiology Study (KoGES) was initiated in 2001. It included a total of 10,038 participants aged 40–69 years. The cohort consisted of baseline data from 2001 to 2002 and follow-up data from 2003–2014 (every two years). Among them, secondary follow-up data between 2005 and 2006 that included metabolite information were selected for this study. A total of 2579 participants with both metabolomic and genomic data were available. Basic characteristics of the participants are provided in Table 1.

All participants underwent anthropometric and biochemical measurements. Information about these measurements is included in the KARE dataset. Height and weight values were obtained through an automated measuring instrument (Dong Sahn Jenix Co., Seoul, Republic of Korea). Blood pressure measurements were performed three times at intervals of more than five minutes in a sitting position, using a mercury sphygmomanometer (Baumanometer; W.A. Baum, Copiague, NY, USA). Their average values were used in this study. Blood sampling was performed to analyze biochemical traits such as serum creatinine and blood urea nitrogen (BUN) in individuals who fasted for more than 8 h. Serum creatinine was determined via the Jaffe method using an automatic analyzer (Hitachi, Tokyo, Japan) [21]. The CKD-Epidemiology Collaboration (CKD-EPI) equation was used to calculate the eGFR. The eGFR equations used in this study according to gender are described below:

eGFR in Men = 141 × min (creatinine/0.9, 1)^−0.411^ × max (creatinine/0.9, 1)^−1.209^ × 0.993^age^

eGFR in Women = 143.54 × min (creatinine/0.7, 1)^−0.329^ × max (creatinine/0.7, 1)^−1.209^ × 0.993^age^

For case-control analysis, participants were divided into two groups (CKD and non-CKD) based on the criteria of the Kidney Disease Improving Global Outcome (KDIGO) [22]. The CKD group (*n* = 264) consisted of people with eGFR < 60 mL/min/1.72 m^2^ and a history of CKD. The non-CKD group (*n* = 1550) was defined as an eGFR ≥ 60 mL/min/1.72 m^2^. Patients (*n* = 765) with a diagnosis or drug history of diabetes and/or hypertension were excluded from the non-CKD group.

### 2.2. Metabolite Measurements

Serum metabolite quantification for 2579 participants was carried out using an AbsoluteIDQ p180 kit (BIOCRATES Life Science, Innsbruck, Austria) following the manufacturer’s instructions. An API 4000 QTRAP system (Applied Biosystems, Foster City, CA, USA) equipped with an Agilent 1200 HPLC system (Agilent Technologies, Santa Clara, CA, USA) was used to perform liquid chromatography and flow injection analysis-mass spectrometry. Concentrations of kynurenine and tryptophan used in this study were measured in μM units with a MetVal software package (BIOCRATES Life Sciences). Serum metabolites that passed the following quality control (QC) criteria were included in this study: (i) the coefficient of variance for each metabolite in the reference standards < 25%, (ii) half of the analyzed metabolite concentrations in the reference standards > limit of detection (LOD), and (iii) half of the analyzed metabolite concentrations in the experimental samples > LOD. Detailed information for the QC procedure has been described in a previous study [23]. The ratio of kynurenine to tryptophan (K/T) was calculated to estimate the IDO activity.

### 2.3. Genotyping and Imputation

Genomic DNA samples were extracted from peripheral blood samples and genotyped using an Affymetrix Genome-Wide Human SNP array 5.0 (Affymetrix, Santa Clara, CA, USA). Detailed QC procedures for samples and single nucleotide polymorphisms (SNPs) have been previously reported [24]. Briefly, samples were excluded if they had high missing call rates, ≥ 4%, DNA contamination, sex inconsistencies, or cryptic relatedness. Genotyping data were cleaned according to QC criteria [excluding SNPs with missing call rates > 0.05, Hardy-Weinberg equilibrium (HWE) *p*-value < 1 × 10^−6^, or minor allele frequency (MAF) < 0.01]. The imputation of SNPs was performed using the 1000 Genomes Phase I data (reference panel) and the IMPUTE2 software [25]. Finally, 6,461,358 SNPs were included in the current analysis. Locations of SNPs were based on the National Center for Biotechnology Information (NCBI) Human Genome Build 37 (hg19).

### 2.4. Statistical Analysis

Regression analyses based on the additive model were conducted using PLINK version 1.90 beta (https://www.cog-genomics.org/plink2 (accessed on 10 March 2023)) [26]. A student’s *t*-test was used to compare the mean values of several characteristics between cases and controls. Quantitative phenotypes such as IDO and eGFR were analyzed via linear regression. Logistic regression was used to test the association between SNPs and CKD. *p*-values were adjusted for covariates such as age, sex, geographic area, body mass index (BMI), hemoglobin A1C (HbA1c), alcohol intake, smoking, systolic blood pressure (SBP), and high-sensitivity C-reactive protein (hs-CRP). We selected SNPs that showed *p* < 0.001 for both IDO activity and CKD. To avoid enrichment testing for multiple SNPs showing the same genetic linkage signal, SNPs were clumped for linkage disequilibrium (LD) with an *r*^2^ threshold 0.5 within a distance of 1000 kb. Regional plots were generated with the LocusZoom browser (http://locuszoom.org/ (accessed on 10 March 2023)). *In silico* analysis was performed using genotype tissue expression (GTEx) (https://gtexportal.org/ (accessed on 10 March 2023)) and HaploReg v4.1 (https://pubs.broadinstitute.org/ (accessed on 10 March 2023)) databases. The transforming growth factor-β (TGF-β) signaling pathway was downloaded from the Kyoto Encyclopedia of Genes and Genomes (KEGG) pathway website (https://www.genome.jp/kegg/ (accessed on 2 February 2023)).

### 2.5. Genotype-Tissue Expression (GTEx)

The GTEx portal is a data resource and tissue bank established by the National Institutes of Health Common Fund to investigate the correlation between genetic variation and gene expression in multiple tissues [27]. The donors comprise nearly 1000 individuals who have consented to donate their organs and/or tissues after death, including various ethnicities such as European American, African American, Asian American, Hispanic, or Latino. The samples were collected from 54 non-diseased tissue sites (such as brain, thyroid, pancreas, skin, etc.) and 2 cell lines. Detailed information about tissue sampling sites can be found on the GTEx portal (https://gtexportal.org/ (accessed on 10 March 2023)).

### 2.6. Ethics Statement

This study was approved by the Institutional Review Board (IRB) of the Korea Disease Control and Prevention Agency (KDCA) (approval number: KBN-2021-003; date: 26 January 2021) and Soonchunhyang University (approval number: 202012-BR-086-01; date: 15 December 2020). All participants provided written informed consent.

## 3. Results

### 3.1. Characteristics of Study Participants

The clinical profiles of the study population are summarized in Table 1. The average age of 2579 participants was 57.10 ± 9.05 years. There were 1218 (47.23%) males. Compared with healthy controls (*n* = 1550), CKD patients (*n* = 264) were older with higher BMI, creatinine, and BUN levels. In contrast, height, weight, and eGFR were lower in the case group than in the control group. All characteristics were significantly (*p* < 0.05) different between the case group and the control group.

### 3.2. Variants Associated with Both IDO Activity and CKD

Using regression analysis, this study performed genome-wide analysis to investigate associations of SNPs with IDO activity and CKD. As a result, we identified 10 tag SNPs that were significantly associated with both IDO activity and CKD (*p* < 0.001) (Table 2). Of these, rs189138212 located near the *ITPKB* (Inositol-Trisphosphate 3-Kinase B) gene showed the highest association (*p* = 7.19 × 10^−5^) with IDO activity. However, its frequency was too low (MAF = 0.017). In addition, no strong LD was observed in the vicinity (Figure S2). Therefore, we first excluded genes irrelevant to IDO activity and CKD as well as variants showing weak LD values with surrounding SNPs to select candidate genes/loci. Eventually, we focused on rs6550842, rs77624055, and rs35651150 located at 3p24.2, 6p24.3, and 8p21.3, respectively.

**Figure 1:**
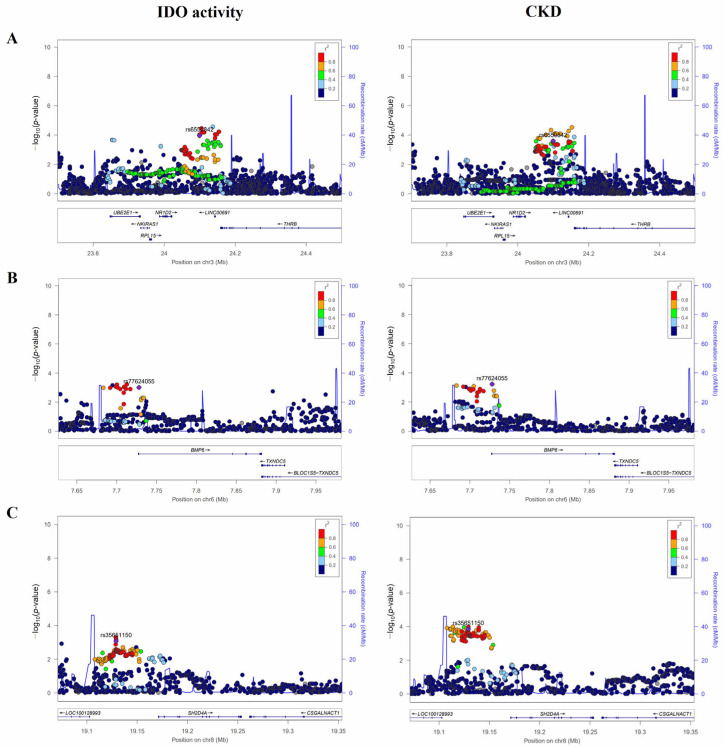
Regional association plots of IDO activity and CKD at 3p24.2 (**A**), 6p24.3 (**B**) and 8p21.3 (**C**) loci. Statistical significances of SNPs near *NKIRAS1*, *BMP6*, and *SH2D4A* for IDO activity and CKD are plotted as −log_10_ *p*-values. The purple diamond (◆) represents the SNP strongly involved in both CKD and IDO activity. Levels of linkage disequilibrium (*r*^2^) of top SNPs and surrounding SNPs are shown in different colors. These regional plots for SNPs were generated via LocusZoom(http://locuszoom.org/ (accessed on 10 March 2023)). IDO, indoleamine 2,3-dioxygenase; CKD, chronic kidney disease.

The recombination rates (cM/Mb) and LD values of three candidate variants are shown in regional plots (Figure 1). Locus 3p24.2 reached high significance levels with *p* = 1.07 × 10^−4^ and *p* = 2.52 × 10^−4^ for rs6550842 in IDO activity and CKD, respectively. The LD block of this variant was surrounded by several genes (*UBE2E1*, Ubiquitin Conjugating Enzyme E2 E1; *NKIRAS1*, NFKB Inhibitor Interacting Ras Like 1; *RPL15*, Ribosomal Protein L15; *NR1D2*, Nuclear Receptor Subfamily 1 Group D Member 2; *LINC00691*, Long Intergenic Non-Protein Coding RNA 691; *THRB*, Thyroid Hormone Receptor Beta) on chromosome 3. The second associated region at 6p24.3 also showed significant associations with IDO activity and CKD (*p* = 9.70 × 10^−4^ and *p* = 5.89 × 10^−4^ for rs77624055, respectively) rs77624055 is a variant located in the intronic of the *BMP6* (Bone Morphogenetic Protein 6) gene. The rs35651150 of the third locus at 8p21.3 is an intergenic SNP located 26 kb upstream of *LOC100128993* and 42 kb upstream of *SH2D4A* (SH2 Domain Containing 4A). Its minor allele carriers significantly reduced both IDO level (*β* = −0.11, *p* = 8.30 × 10^−4^) and CKD risk (OR = 0.58, 95% CI: 0.44–0.77, *p* = 1.37 × 10^−4^). All these tag SNPs (rs6550842, rs77624055, and rs35651150) also showed significant associations with eGFR (all *p* < 0.05).

Since rs6550842 and rs35651150 are located between several genes rather than belonging to one specific gene, we further analyzed rs77624055 located in the intron of the *BMP6* gene. SNPs within the same region (7.68–7.74 kb at 6p24.3) as rs77624055, based on recombination rates, were included in the analysis. Five out of 16 tag SNPs, including rs77624055, reached a statistical significance of *p* < 0.05 for associations with IDO activity (Table 3). These variants had signals independent of each other. Among them, rs7753111 showed statistically significant associations with IDO activity (*β* = 0.085, *p* = 5.56 × 10^−3^), CKD (OR = 1.31, 95% CI 1.04–1.66, *p* = 0.024), and eGFR (*β* = −0.74, *p* = 0.019). Additionally, rs111588693 in the *BMP6* gene was associated with IDO activity (*β* = −0.085, *p* = 0.023) as a non-synonymous variant.

### 3.3. In Silico Analysis

**Figure 2:**
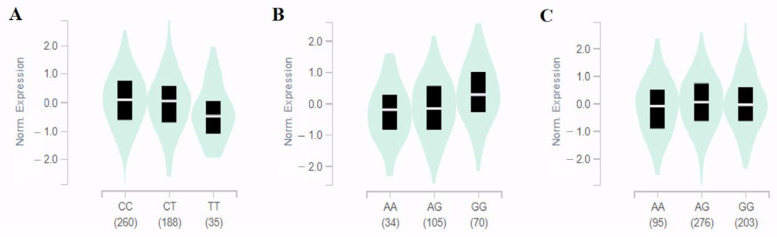
Identification of *NKIRAS1* and *SH2D4A* expression and rs6550842 (**A**) and rs35651150 (**B**,**C**) in eQTL, respectively. Violin plots show *NKIRAS1* expression in cultured fibroblasts for the rs6550842 genotype ((**A**), effect size = −0.12, *p* = 7.3 × 10^−5^). The *SH2D4A* expression for the rs35651150 genotype in the cerebellum ((**B**), effect size = 0.37, *p* = 1.3 × 10^−5^) and thyroid ((**C**), effect size = 0.19, *p* = 7.7 × 10^−9^) was confirmed and statistically significant. Mean expression levels are indicated by the white line and 25% and 75% quantiles are shown by the black box. Data were obtained via GTEx browser (https://gtexportal.org/ (accessed on 10 March 2023)).

This study performed eQTL analysis based on the GTEx database to determine gene expression levels according to SNP genotypes. eQTL was found for two SNPs except for rs77624055 in human tissues (Figure 2). As a result, the minor allele (A) of rs35651150 located between *LOC100128993* and *SH2D4A* contributed to lower expression levels of the *SH2D4A* gene in the cerebellum (effect size = 0.37, *p* = 1.3 × 10^−5^) and thyroid (effect size = 0.19, *p* = 7.7 × 10^−9^) (Figure 2B,C) respectively. In particular, rs6550842 only affected the expression of the *NKRAS1* gene, although it was located between several genes (*NKIRAS1*-*NR1D2*-*THRB*).

In cultured fibroblasts, the expression level of the *NKIRAS1* gene was significantly reduced in minor allele (T) carriers of rs6550842 (effect size = −0.12, *p* = 7.3 × 10^−5^) (Figure 2A). Thus, its true functional association was believed to be with *SH2D4A* for rs35651150 and *NKIRAS1* for rs6550842, respectively. For the *BMP6* gene without eQTL data, potential functional effects of five SNPs related to IDO activity were examined using the HaploReg database (Table S1). Motif changes were predicted for the four SNPs that showed significant associations with IDO activity. The TGF-β signaling pathway involved in many cellular processes is also shown through KEGG pathway analysis of the *BMP6* gene (Figure S1).

## 4. Discussion

Various populations, including Koreans [28], have provided evidence to support the connection between IDO activity and CKD [7,13,14]. We have recently conducted an integrated metabolome and genome association analysis to identify genetic markers for detecting CKD in its early stages [15]. However, the previous study that focused on IDO activity had a limitation, as the correlation with CKD was moderately low. To overcome this issue, we conducted the present study to identify the causative gene without any bias towards a specific phenotype. As a result, we identified 10 SNPs showing significant associations with both IDO activity and CKD (Table 2). Among them, four SNPs (rs189138212, rs145478425, rs149583220, and rs145951089) showed low LD values with surrounding SNPs in their respective genetic regions, suggesting that their genetic signals were weak (Figures S2 and S3). Also, rs7679032, rs9533960, and rs72898186 were considered signals unrelated to IDO activity or CKD. Eventually, we selected three candidate genes/loci (*NKIRAS1*/3p24.2, *BMP6*/6p24.3, and *SH2D4A*/8p21.3), excluding unrelated and weak signals.

Among these three selected variants, rs6550842 is an intergenic tag SNP located between several genes (*UBE2E1*, *NKIRAS1*, *RPL15*, *NR1D2*, *LINC00691*, and *THRB*), and rs35651150 is located between the *LOC100128993* and *SH2D4A* genes. Previous studies have reported that eQTL analysis can be used to identify novel trait-related loci and determine the most functionally related genes at loci [18,29]. Therefore, we performed eQTL analysis to identify target genes. eQTL analysis shows that rs6550842 affected the expression of the *NKIRAS1* gene in cultured fibroblasts (Figure 2A). Additionally, although rs35651150 was closer to the *LOC100128993* gene, it significantly altered the *SH2D4A* gene expression levels in the cerebellum and thyroid (Figure 2B,C). Based on some evidence indicating their connections with IDO activity and CKD, we further investigated the *NKIRAS1* and *SH2D4A* genes, as described below.

The first candidate gene, *NKIRAS1*, encodes a Ras-like protein that has a close association with the NF-kappa-B (NF-κB) pathway [30]. Activation of NF-κB, which controls many genes involved in inflammation, has been reported to play an important role in the inflammatory response of kidney injury [31,32]. In light of these studies, *NKIRAS1*, which negatively regulates NF-κB activity [30], can improve renal impairment. Indeed, Gerashchenko et al. have reported that the expression of *NKIRAS1* is downregulated in renal cell carcinoma [33]. Consistent with previous findings, our results showed that being a minor carrier of rs6550842 decreased the expression of *NKIRAS1* but increased both CKD risk and IDO activity (Table 2 and Figure 2).

As a second candidate, the *BMP6* gene encodes a protein which is a member of the TGF-β superfamily [34]. The TGF-β is a potent fibrotic cytokine involved in many cellular processes including cell growth, differentiation, and migration [35]. Many studies have found increased expression and activation of TGF-β1 in progressive kidney disease [35,36,37]. In addition, TGF-β1, a potent fibrogenic cytokine, has been reported to regulate IDO in some cell types [38]. Remarkably, in vivo studies have confirmed that deficiency of the *BMP6* gene can increase renal fibrosis and upregulate TGF-β1 after renal injury [39,40,41]. TGF-β is known to activate the Smad2/3 and c-Jun NH2-terminal kinase (JNK) signaling pathways [42]. Further to this discovery, Yan et al. have conducted cell experiments and suggested that inhibition of JNK and Smad2/3 signaling activated by TGF-β1 is a major mechanism for the antifibrotic effect of the *BMP6* gene [43].

In this study, the minor allele of rs77624055 in the *BMP6* gene showed the same tendency to increase both IDO activity and CKD risk (Table 2). In combination with several previous studies, our results suggest that the *BMP6* gene might ultimately be involved in CKD by regulating renal fibrosis as well as IDO activity via the TGF-β signaling pathway (Figure S1). On the other hand, since the currently available eQTL database does not provide evidence for rs77624055, further functional studies are needed to elucidate whether this variant affects the expression of the *BMP6* gene associated with IDO and CKD.

The estrogen receptor α (ERα) is a nuclear transcription factor involved in physiological development and function in humans [44]. Regulating its activity might be useful for preventing and treating various diseases. Previous studies have reported that estrogen and the ERα are involved in kidney repair and regeneration [45,46]. As a final candidate gene, the *SH2D4A* gene encodes a protein that belongs to the SH2 signaling protein family, which has been reported to bind directly to the ERα in kidney cells [47]. Accordingly, we hypothesize that SH2D4A, which inhibits the ER signaling pathway, could potentially have adverse effects on the kidneys. Consistent with our hypothesis, minor allele carriers of rs35651150 had decreased IDO activity and CKD risk, as well as downregulated expression levels of the *SH2D4A* gene in human tissues (Table 2 and Figure 2). These observations provide evidence that the *SH2D4A* gene, associated with the ERα, affects IDO activity and CKD.

The limitation of this study is that the sample size was insufficient to perform separate analyses for males and females. Additionally, the absence of parental information in the KARE cohort posed a challenge in establishing a cause-and-effect relationship between family history of kidney disease and IDO activity. Therefore, further replication studies in other cohorts are necessary to validate our findings and investigate the impact of gender.

In a previous study, we demonstrated that the regulation of IDO activity by four genes (*RSU1*, *PDGFD*, *SNX25* and *TNFRSF19*) is linked to CKD. These findings could aid in the early diagnosis of CKD by utilizing SNPs of these genes to predict IDO activity. In contrast to the previous study, the present research conducted an analysis to find genes that are commonly associated with both CKD and IDO activity. Of the 10 identified SNPs, we focused on rs6550842 (*NKIRAS1*), rs77624055 (*BMP6*), and rs35651150 (*SH2D4A*). Moreover, we confirmed whether identified variants could affect gene expression. As a result, rs6550842 and rs35651150 of *NKIRAS1* and *SH2D4A* genes, respectively, showed important signals in human tissues. In particular, the *NKIRAS1* and *BMP6* genes are implicated in the NF-κB and TGF-β pathways, respectively. This suggests that IDO activity might act on CKD through the NF-κB and TGF-β pathways, which are closely related to inflammation.

## 5. Conclusions

The study suggests that *NKIRAS1*, *BMP6*, and *SH2D4A* could be promising new genetic markers for early improvement in renal health. Additionally, in vivo experiments should be performed to extend the genetic markers identified in this study to the mechanisms of disease physiology.

## Figures and Tables

**Table 1:** Characteristics of participants in this study.

Characteristics	Quantitative Trait Analysis	Case-Control Analysis for CKD		
		Controls	Cases	*p*-Value ^1^
Number of participants	2579	1550	264	
Gender [men/women]	1218/1361	789/761	81/183	<0.001
Age (M years ± SD)	57.10 ± 9.05	54.98 ± 8.64	65.72 ± 6.53	<0.001
Height (M cm ± SD)	159.55 ± 9.16	160.55 ± 8.98	155.42 ± 8.28	<0.001
Weight (M kg ± SD)	62.63 ± 10.36	62.30 ± 10.41	60.88 ± 9.53	0.042
BMI (M kg/m^2^ ± SD)	24.56 ± 3.23	24.11 ± 3.09	25.20 ± 3.47	<0.001
eGFR (mL/min/1.73 m^2^)	75.58 ± 11.92	78.68 ± 9.69	55.24 ± 9.21	<0.001
Creatinine (mg/dL)	0.98 ± 0.20	0.96 ± 0.14	1.18 ± 0.42	<0.001
BUN (mg/dL)	15.69 ± 4.26	15.33 ± 3.92	17.91 ± 5.45	<0.001

^1^ Significant differences in characteristics between cases and controls were determined with Student’s *t*-test. BMI, body mass index; eGFR, estimated glomerular filtration rate; BUN, blood urea nitrogen; CKD, chronic kidney disease; M, mean value; SD, standard deviation.

**Table 2:** Loci associated with both indoleamine 2,3-dioxygenase activity and chronic kidney disease in Koreans.

SNP	Nearest Gene	Chromosome Position	Minor Allele	MAF	Function	IDO Activity		CKD		eGFR	
						*β* ± S.E	*p*-Value	OR (95% CI)	*p*-Value	*β* ± S.E	*p*-Value
rs189138212	*ITPKB*	1:226951229	C	0.017	-	0.44 ± 0.11	7.19 × 10^−5^	3.55 (1.71–7.37)	6.78 × 10^–4^	−3.34 ± 1.13	3.28 × 10^–3^
rs6550842	***NKIRAS1-NR1D2-THRB***	3:24099794	T	0.107	-	0.18 ± 0.05	1.07 × 10^−4^	1.89 (1.34–2.65)	2.52 × 10^–4^	−1.77 ± 0.47	1.76 × 10^–4^
rs145478425	*FHIT*	3:59803424	T	0.015	Intron	0.41 ± 0.12	4.04 × 10^−4^	3.63 (1.78–7.40)	3.95 × 10^–4^	−1.26 ± 1.20	0.293
rs7679032	*LINC00616*	4:138949061	T	0.031	Intron	0.32 ± 0.08	1.26 × 10^−4^	2.65 (1.53–4.60)	5.24 × 10^–4^	−1.32 ± 0.85	0.120
rs77624055	***BMP6***	6:7727931	A	0.167	Intron	0.13 ± 0.04	9.70 × 10^−4^	1.67 (1.25–2.23)	5.89 × 10^–4^	−0.93 ± 0.39	0.017
rs35651150	***SH2D4A***	8:19129080	A	0.256	-	−0.11 ± 0.03	8.30 × 10^−4^	0.58 (0.44–0.77)	1.37 × 10^–4^	1.16 ± 0.33	4.48 × 10^–4^
rs149583220	*ADAM7*	8:24307595	C	0.011	Intron	0.48 ± 0.13	2.99 × 10^−4^	4.64 (2.06–10.44)	2.16 × 10^–4^	−4.26 ± 1.36	1.75 × 10^–3^
rs145951089	*LOC101927318*	12:50355316	T	0.026	Intron	0.34 ± 0.09	1.40 × 10^−4^	3.13 (1.71–5.73)	2.15 × 10^–4^	−2.43 ± 0.91	7.80 × 10^–3^
rs9533960	*LINC00330*	13:45333274	C	0.320	-	0.11 ± 0.03	5.04 × 10^−4^	1.51 (1.19–1.92)	7.31 × 10^–4^	−0.63 ± 0.31	0.041
rs72898186	*MIR924HG*	18:37071519	C	0.163	Intron	0.13 ± 0.04	9.80 × 10^−4^	1.72 (1.29–2.29)	2.49 × 10^–4^	−0.75 ± 0.39	0.056

SNP, single nucleotide polymorphism; MAF, minor allele frequency; IDO, indoleamine 2,3-dioxygenase; CKD, chronic kidney disease; eGFR, estimated glomerular filtration rate; *β*, regression coefficient; S.E, standard error; OR, odds ratio; CI, confidence interval. All analyses were adjusted for age, sex, area, BMI, alcohol intake, smoking, SBP, hs-CRP and HbA1c. SNPs that achieved *p* < 0.001 in both IDO activity and CKD were selected. Locations of SNPs were based on the National Center for Biotechnology Information (NCBI) Human Genome Build 37 (hg19). Genes stated in the manuscript are indicated in bold.

**Table 3:** Association results of SNPs near *BMP6* gene with IDO activity, CKD, and eGFR.

SNP	Chromosome Position	Minor Allele	MAF	Function	IDO Activity		CKD		eGFR	
					*β* ± S.E	*p*-Value	OR (95% CI)	*p*-Value	*β* ± S.E	*p*-Value
**rs77624055**	7727931	A	0.167	Intron	0.125 ± 0.038	**9.70 × 10^–4^**	1.67 (1.25–2.23)	**5.89 × 10^−4^**	−0.93 ± 0.39	**0.017**
**rs7753111**	7730944	G	0.299	Intron	0.085 ± 0.031	**5.56 × 10^–3^**	1.31 (1.04–1.66)	**0.024**	−0.74 ± 0.31	**0.019**
**rs2224564**	7737187	C	0.297	Intron	0.082 ± 0.031	**8.99 × 10^–3^**	0.93 (0.73–1.19)	0.564	−0.57 ± 0.32	0.079
**rs111588693**	7727271	A	0.179	Non-synonymous (R28Q)	−0.085 ± 0.038	**0.023**	0.89 (0.65–1.23)	0.484	0.06 ± 0.38	0.875
rs76295967	7733744	A	0.009	Intron	−0.305 ± 0.151	**0.043**	0.71 (0.14–3.52)	0.676	0.34 ± 1.54	0.826
rs7766858	7694908	G	0.478	-	0.043 ± 0.029	0.132	1.32 (1.05–1.65)	**0.015**	−0.60 ± 0.29	**0.041**
rs270407	7738392	C	0.478	Intron	−0.043 ± 0.028	0.132	1.21 (0.97–1.52)	0.090	0.13 ± 0.29	0.657
rs962279	7708132	G	0.324	-	−0.043 ± 0.030	0.150	0.95 (0.74–1.22)	0.690	−0.05 ± 0.31	0.859
rs1923409	7728212	A	0.370	Intron	0.031 ± 0.029	0.297	1.25 (0.99–1.58)	0.057	−0.59 ± 0.30	**0.048**
rs270417	7729614	C	0.025	Intron	−0.095 ± 0.091	0.297	1.68 (0.84–3.36)	0.145	−0.63 ± 0.93	0.498
rs9505263	7698011	T	0.017	-	−0.106 ± 0.109	0.329	0.65 (0.26–1.65)	0.364	0.64 ± 1.11	0.565
rs79982308	7691593	A	0.016	-	0.068 ± 0.110	0.535	1.29 (0.60–2.75)	0.513	−0.16 ± 1.12	0.886
rs75686372	7737224	T	0.016	Intron	0.060 ± 0.115	0.603	1.84 (0.88–3.85)	0.105	−1.05 ± 1.18	0.371
rs118168182	7735363	T	0.028	Intron	0.027 ± 0.088	0.759	0.95 (0.49–1.86)	0.888	1.52 ± 0.89	0.090
rs79560447	7690928	T	0.142	-	−0.005 ± 0.042	0.902	0.77 (0.56–1.07)	0.115	0.27 ± 0.42	0.526
rs2876117	7738931	T	0.138	Intron	0.000 ± 0.040	0.994	0.91 (0.67–1.25)	0.573	0.27 ± 0.41	0.507

SNP, single nucleotide polymorphism; MAF, minor allele frequency; IDO, indoleamine 2,3-dioxygenase; CKD, chronic kidney disease; eGFR, estimated glomerular filtration rate; *β*, regression coefficient; S.E, standard error; OR, odds ratio; CI, confidence interval. All analyses were adjusted for age, sex, geographic area, BMI, alcohol intake, smoking, SBP, hs-CRP, and HbA1c. The locations of SNPs were based on the National Center for Biotechnology Information (NCBI) Human Genome Build 37 (hg19). Genes stated in the manuscript are indicated in bold.

## Data Availability

The data presented in this study are available on request from the corresponding author. The data are not publicly available due to ethnical concerns.

## Supplementary Materials

The following supporting information can be downloaded at: https://www.mdpi.com/article/10.3390/metabo13040541/s1, Figure S1: The KEGG pathway for TGF-*β* signaling pathway; Figure S2: Regional plots for association of SNPs near *ITPKB* (A), *LOC101927318* (B), and *LINC00330* (C) genes; Figure S3: Regional plots for association of SNPs near *ADAM7* (A), *FHIT* (B), and *LINC00616* (C), *MIR924HG* (D) genes; Table S1: HaploReg results of four SNPs in the *BMP* gene.
