# Supplementary material for: Identification of Genetic Markers Linked to The Activity of Indoleamine 2,3-Dioxygenase and Kidney Function
Source: Metabolites. 2023 Apr 10;13(4):541. doi: 10.3390/metabo13040541 (PMC10144659; doi:10.3390/metabo13040541)
Supplement: Supplementary file 1 [file metabolites-13-00541-s001.zip › metabolites-2308554-Supplementary Materials.pdf]

metabolites

MDPI

Supplementary Figures

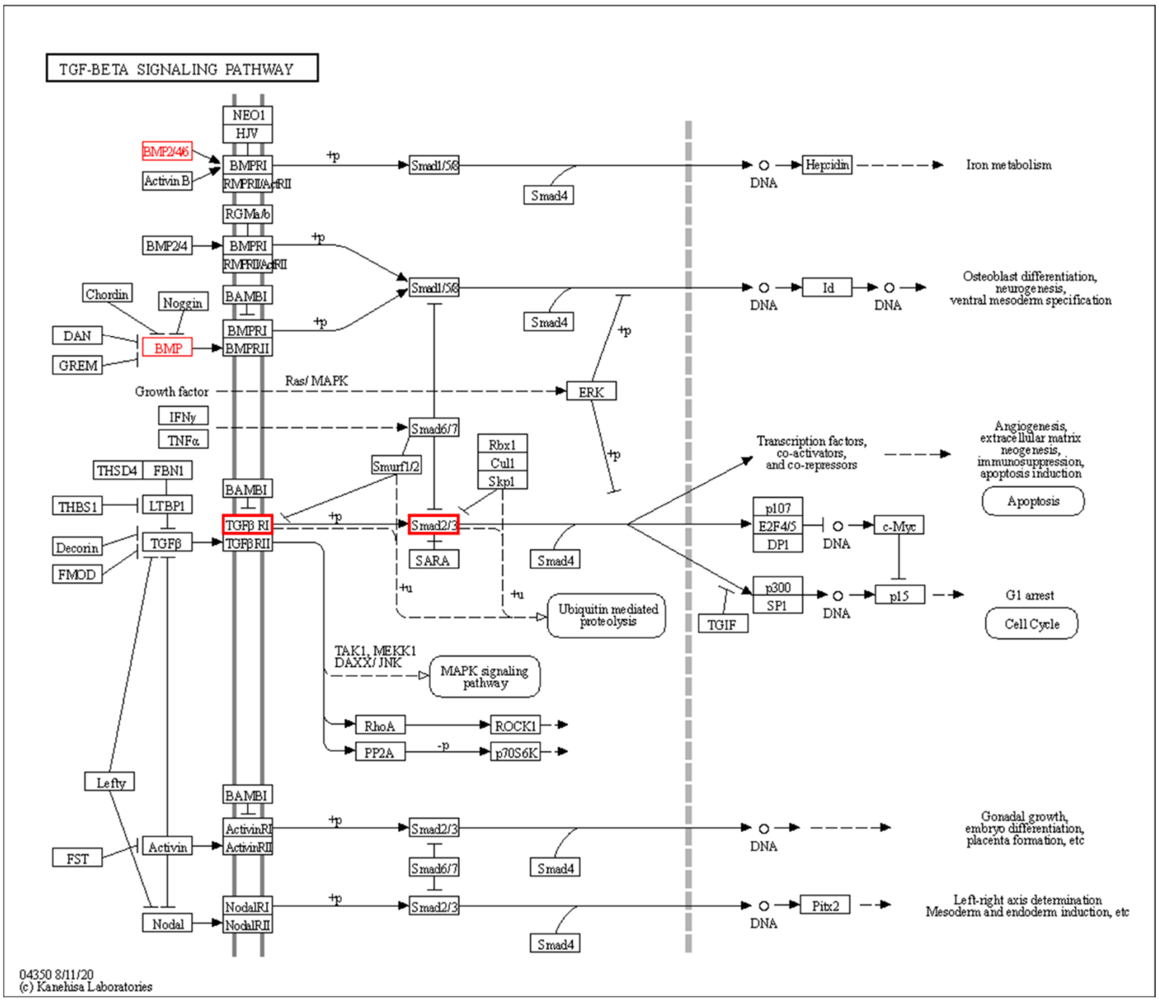

Figure S1. The KEGG pathway for TGF-β signaling pathway. A red box indicates the *BMP6* gene according to KEGG. The image of a pathway was generated via KEGG browser (https://www.genome.jp/kegg/ (accessed on 2 February 2023)). TGF-β signaling pathway is closely connected with chronic kidney disease.

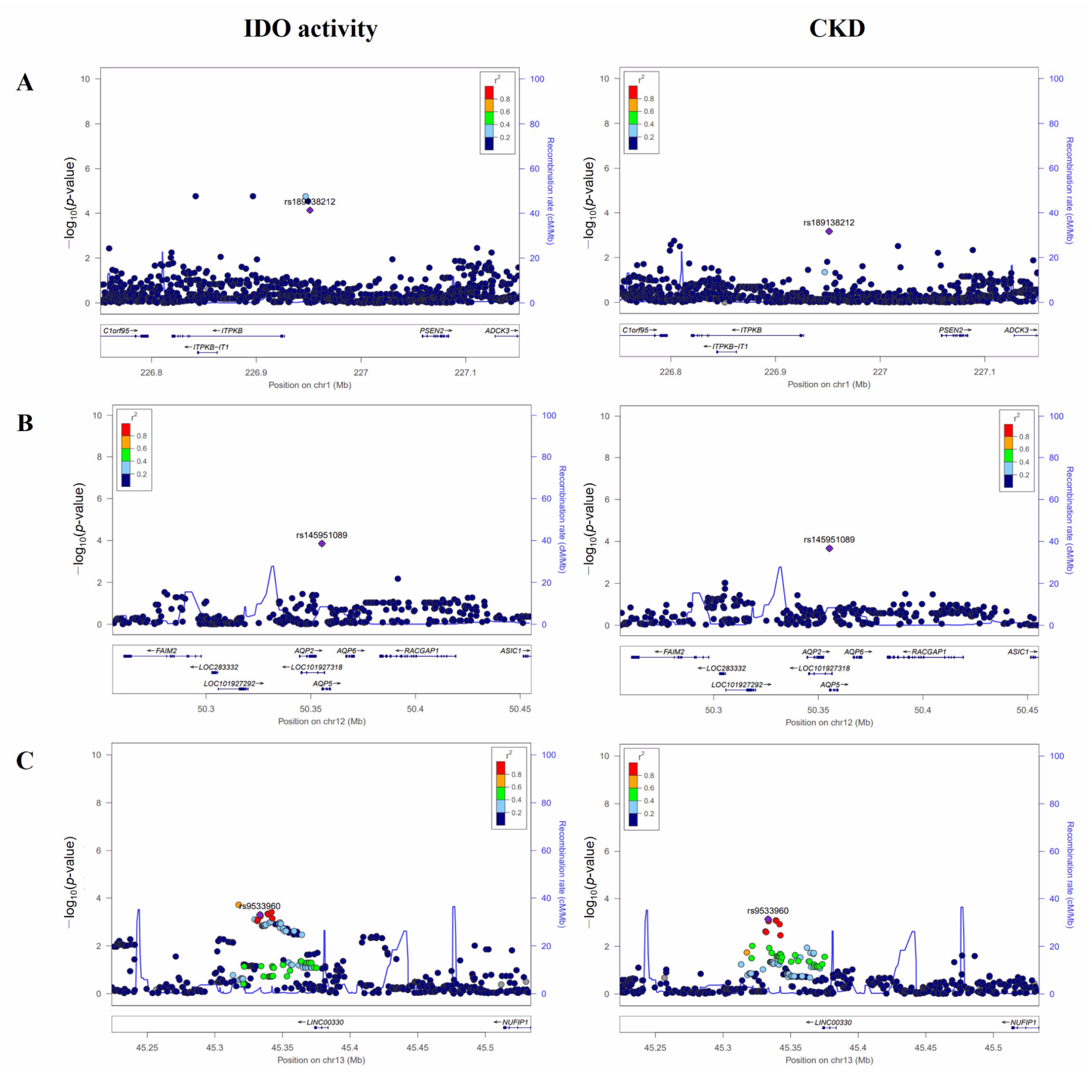

Figure S2. Regional plots for association of SNPs near *ITPKB* (a), *LOC101927318* (b), and *LINC00330* (c) genes. Statistical significances of SNPs for IDO activity and CKD are plotted as $-\log_{10}P$ values. The purple diamond represents the SNP strongly involved in both IDO activity and CKD. Levels of linkage disequilibrium (r^2) of marked SNPs and surrounding SNPs are shown in different colors. These regional plots for SNPs were generated via LocusZoom browser (http://locuszoom.org/ (accessed on 10 March 2023)).

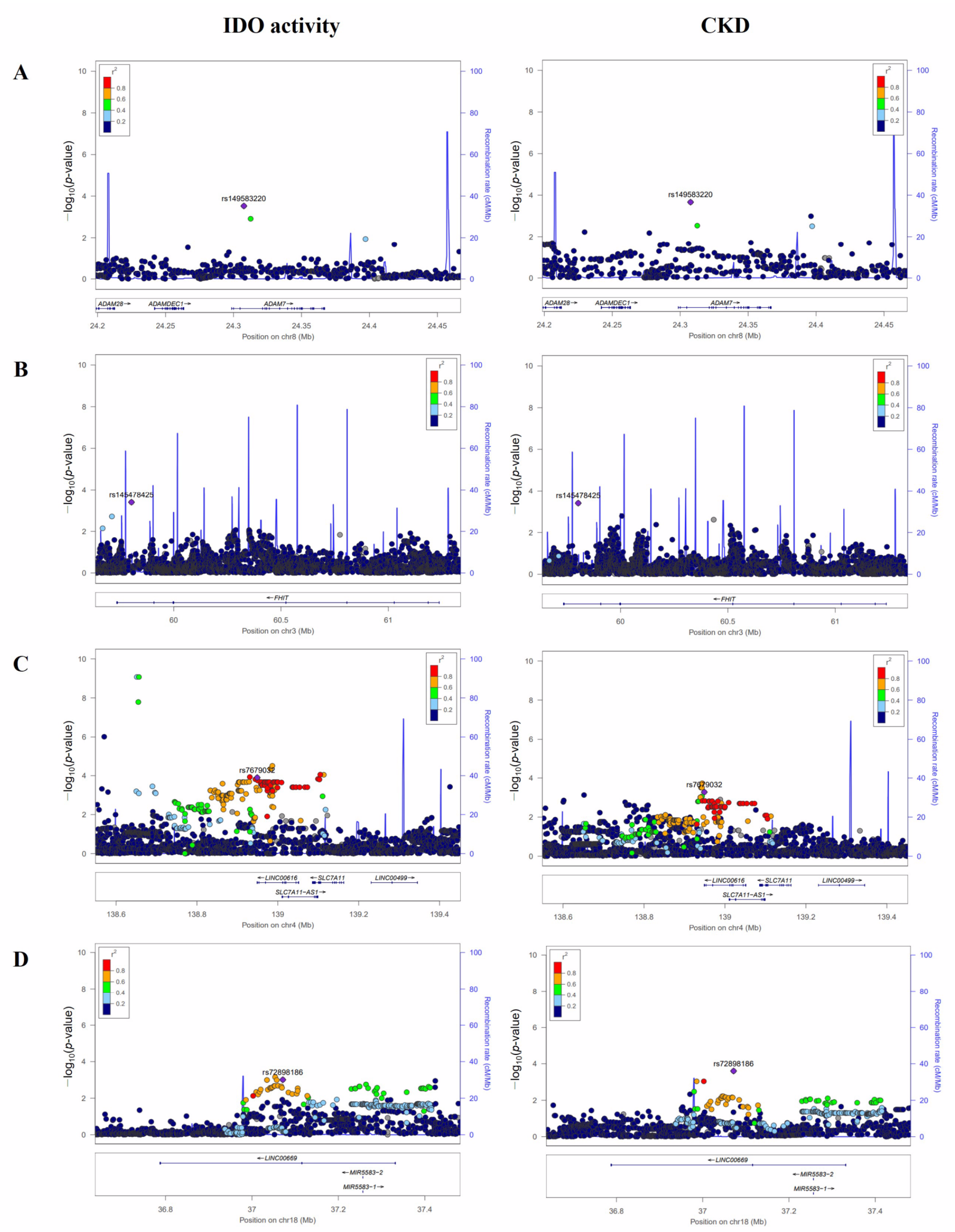

Figure S3. Regional plots for association of SNPs near *ADAM7* (a), *FHIT* (b), and *LINC00616* (c), *MIR924HG* (d) genes. Statistical significances of SNPs for IDO activity and CKD are plotted as $-\log_{10}P$ values. The purple diamond represents the SNP strongly involved in both IDO activity and CKD. Levels of linkage disequilibrium (r^2) of marked SNPs and surrounding SNPs are shown in different colors. These regional plots for SNPs were generated via LocusZoom browser (http://locuszoom.org/ (accessed on 10 March 2023)).

Supplementary Table

Table S1 HaploReg results of four SNPs in the BMP gene.

SNP	Minor allele	HaploReg[1]				
		Promoter histone markers	Enhancer histone markers	DNase	Proteins bound	Motifs
rs77624055	G	20 tissues	10 tissues	6 tissues	HAE2F1, INI1, ZNF263	13 altered motifs
rs7753111	A	5 tissues	11 tissues	ESDR, CRVX, VAS	CEBPB, JUND, P300	-
rs2224564	C	-	VAS	-	-	GLI, Zfx
rs111588693	G	20 tissues	11 tissues	-	-	12 altered motifs
rs76295967	A	-	BLD, FAT, LIV	VAS	-	16 altered motifs

[1] HaploReg: https://pubs.broadinstitute.org/mammals/haploreg/haploreg.php (accessed on 10 March 2023). Abbreviations: SNP, single nucleotide polymorphism.
